# Subgenual cingulate connectivity and hippocampal activation are related to MST therapeutic and adverse effects

**DOI:** 10.1038/s41398-020-01042-7

**Published:** 2020-11-10

**Authors:** Itay Hadas, Reza Zomorrodi, Aron T. Hill, Yinming Sun, Paul B. Fitzgerald, Daniel M. Blumberger, Zafiris J. Daskalakis

**Affiliations:** 1 Department of Psychiatry, Faculty of Health, University of California San Diego, La Jolla, CA 92093-0603 USA; 2 Temerty Centre for Therapeutic Brain Intervention, Centre for Addiction and Mental Health, Toronto, ON M5T1R8 Canada; 3 Cognitive Neuroscience Unit, School of Psychology, Deakin University, Melbourne, VIC Australia; 4 Department of Psychiatry and Behavioral Sciences, Stanford University School of Medicine, Stanford, CA 94305 USA; 5 Epworth Centre for Innovation in Mental Health, Epworth Healthcare and Monash University Department of Psychiatry, Camberwell, VIC Australia; 6 Department of Psychiatry and Institute of Medical Science, Faculty of Medicine, University of Toronto, Toronto, ON Canada

**Keywords:** Diagnostic markers, Depression

## Abstract

Aberrant connectivity between the dorsolateral prefrontal cortex (DLPFC) and the subgenual cingulate cortex (SGC) has been linked to the pathophysiology of depression. Indirect evidence also links hippocampal activation to the cognitive side effects of seizure treatments. Magnetic seizure therapy (MST) is a novel treatment for patients with treatment resistant depression (TRD). Here we combine transcranial magnetic stimulation with electroencephalography (TMS-EEG) to evaluate the effects of MST on connectivity and activation between the DLPFC, the SGC and hippocampus (Hipp) in patients with TRD. The TMS-EEG was collected from 31 TRD patients prior to and after an MST treatment trial. Through TMS-EEG methodology we evaluated significant current scattering (SCS) as an index of effective connectivity between the SGC and left DLPFC. Significant current density (SCD) was used to assess activity at the level of the Hipp. The SCS between the SGC and DLPFC was reduced after the course of MST (*p* < 0.036). The DLPFC-SGC effective connectivity reduction correlated with the changes in Hamilton depression score pre-to-post treatment (R = 0.46; *p* < 0.031). The SCD localized to the Hipp was reduced after the course of MST (*p* < 0.015), and the SCD change was correlated with montreal cognitive assessment (MOCA) scores pre-post the course of MST (R = −0.59; *p* < 0.026). Our findings suggest that MST treatment is associated with SGC-DLPFC connectivity reduction and that changes to cognition are associated with Hipp activation reduction. These findings demonstrate two distinct processes which drive efficacy and side effects separately, and might eventually aid in delineating physiological TRD targets in clinical settings.

## Introduction

Major depressive disorder (MDD) is a common debilitating condition and is a leading cause of disease burden worldwide. The increasing burden of MDD is associated with considerable morbidity and mortality^1,2^. Up to 40% of patients with MDD do not remit after second line treatment leading to treatment resistant depression (TRD)^3^. To date, electroconvulsive therapy (ECT) is considered to be the most effective treatment for TRD^4^. However, the use of ECT is constrained by multiple factors including patient fears related to the treatment’s cognitive side effects. Therefore, alternative treatment options including magnetic seizure therapy (MST) are being investigated for wider clinical use. To date, open label MST trials have reported similar therapeutic efficacy to ECT with a better cognitive side effect profile further evaluation of MST in clinical settings may benefit from neurophysiological evidence linking MST efficacy and side effects to underlying biological mechanisms.

The subgenual cingulate cortex (SGC) is extensively implicated in the pathophysiology of MDD. There are evidence from studies utilizing positron emission tomography (PET)^5–7^, functional magnetic resonance imaging (fMRI)^8–12^, electroencephalography (EEG)^13^, and postmortem assessments^14^ which closely tie the SGC with the pathophysiology of MDD. The SGC grey matter volume, PET cerebral blood flow and glucose metabolism have been found to be abnormal in MDD patients^6^. PET studies also demonstrated increased blood flow of the SGC co-occurring with instances of sadness and depression, and recovery from depression showed the SGC hyperactivity being normalized^7^. SGC hyperactivity normalization was also demonstrated after different lines of treatments for MDD, such as antidepressant drugs^15–18^, repetitive transcranial magnetic stimulation (rTMS)^13,19,20^ and ECT^21^. These findings led to efforts to treat MDD by implanting deep-brain stimulation electrodes at the SGC white matter^22^. SGC connectivity with the dorsolateral prefrontal cortex (DLPFC) has been found to be associated with depression symptom improvement after rTMS treatment. This association was replicated several times looking at fMRI resting-state functional connectivity measures^8–12^. Moreover, applying TMS-EEG assessment, the MDD symptom improvement was also associated with SGC-DLPFC effective connectivity, and SGC TMS-induced activation^13^.

ECT is the most effective treatment for TRD^4^ and its efficacy has been linked with SGC volume and connectivity changes^23–26^. As mentioned, ECT also induces memory loss^27,28^ which is a significant barrier to patients accepting this very effective treatment^29,30^. MST is a novel seizure inducing, neuromodulatory treatment for MDD with a similar efficacy as ECT^31–33^. By contrast, a large MST clinical trial that included an extensive cognitive battery found that MST had only minimal impact on autobiographical memory consistency^34^. The lack of cognitive side effects after MST is believed to be related to the focality of the induced effective field, and the sparing of hippocampal direct activation during treatment^30,35,36^. To the best of our knowledge, no study has found direct evidence associating the hippocampus (Hipp) with cognitive side effects associated with seizure therapy.

TMS-EEG can be used to derive significant current scattering (SCS) and significant current density (SCD) which assess localized brain connectivity and activation, respectively^37^. Concurrent TMS-EEG allows a temporally and spatially controlled induction of effective electrical field in the brain. Immediately after the TMS pulse the induced brain activation is locally constrained under the coil^38–40^. This activation is then propagated transsynaptically to activate cortical, sub-cortical and eventually peripheral neuronal tissue. Compared to other experimental approaches aimed at evoking brain signal in a time-locked fashion, TMS induced EEG activation, at early post-stimulus latencies (< 100 ms), is independent from sensory and motor inputs and therefore produces a reliable and more anatomically specific activation^38–40^. Moreover, the connectivity metrics TMS-EEG provides are causal, since the initial activation is directly induced by the experimenter, in contrast to functional connectivity metrics which are inferred by non-causal temporal correlations^41^. The SCD computation represents a method to statistically index localized source densities. The SCD can reliably detect source activation by filtering out post-stimulation time frames that failed to present a pronounced activation, due to the probabilistic nature of the neuronal tissue^37,42^. The SCS computation sums the distances between SGC-localized SCD-sources and the TMS target (i.e., the DLPFC)^13,41,43^. The SCS and SCD computations were previously shown to be more robust to spurious activations, and found to be sensitive when applied in studies evaluating MDD patients and repetitive-TMS treatment efficacy^13^, schizophrenia patients^44^, Alzheimer patients^45^, consciousness states^43^, and task-dependent excitability^46^.

Utilizing SCD and SCS computations we aim to test the hypothesis that SGC-DLPFC connectivity is linked to MDD treatment efficacy. We also investigated potential associations between the Hipp and cognitive side effects. Congruous with previously published studies, we hypothesized that SGC-DLPFC effective connectivity would attenuate consistently with MDD symptoms improvement. We also hypothesized that the signal localized to the Hipp would be associated with cognitive changes after the MST trial.

## Methods

### Recruitment and Treatment

The present neurophysiological analysis includes 31 TRD patients (16 male; mean age: 46.13; SD: 11.04) that were recruited at the Centre for Addiction and Mental Health (CAMH; Toronto, Ontario) for an open-label clinical trial of MST (ClinicalTrials.gov Identifier: NCT01596608) (Fig. S1). The patients went through a TMS-EEG recording session, 24-item Hamilton Rating Scale for Depression (HRSD-24) and Montreal Cognitive assessment (MOCA) at baseline and following their final MST treatment. Study protocol was approved by the CAMH research ethics board in accordance with the declaration of Helsinki. Inclusion criteria for this study were: (1) fulfilment of DSM-IV-TR MDD criteria, (2) ECT referral, (3) between the age of 18–85 years, (4) total score >21 according to the HRSD-24, (5) Women of child-bearing potential must be on medically acceptable birth control. Patients Exclusion criteria was: (1) unstable medical/neurological condition, (2) currently pregnant or lactating, (3) insufficiently stable physically to undergo general anesthesia, (4) implanted with any electronic or metallic device or object, (5) use of benzodiazepine, (6) use of anticonvulsant medication, (7) active substance misuse, (8) delirium, dementia or a cognitive disorder secondary to a general medical condition, (9) eating disorder, (10) neuropsychiatric comorbidity, (11) suicide attempt in the last 6 months, (12) diagnosed with antisocial or borderline personality. Patients gave written informed consent, and were treated at the Temerty Centre for Therapeutic Brain Intervention at CAMH. Patients undergoing a course of depression pharmacotherapy were instructed not make changes during the trial. See Table 1 for MDD patient’s demographics and concomitant pharmacotherapy.

**Table. 1:** MDD patients demographics.

*N* = 31	Mean	SD
Age	46.13	11.04
Gender (Male/Female)	16/15	
HRSD-24 Baseline (*n* = 31)	29.05	5.55
HRSD-24 Post (*n* = 22)	19.00	10.43
Number of MST treatments received	18.80	7.40
Responders (%)	45.16%	
MoCA baseline (*n* = 30)	25.55	3.53
MoCA post (*n* = 20)	26.80	2.75
Medications		
Antidepressant	23	
Antipsychotic	9	
Anxiolytics	16	
Stimulants	5	
Lithium	2	

*MDD* major depressive disorder; *HRSD-24* 24-item Hamilton Rating Scale for Depression; *MOCA* Montreal Cognitive Assessment.

### TMS-EEG acquisition

TMS-EEG acquisition for this trial was executed, as previously described (Sun et al., 2016). The TMS-EEG measurements were collected one week before and within two days after the MST treatment course. A 64 channel EEG system (Neuroscan Synamps RT) was used with a 10–20 cap (Neuroscan 64-channels Quik-Cap). The EEG data was recorded at 20 kHz sampling rate and 200 Hz low-pass filter. During the TMS-EEG session a 100 TMS pulses were applied over the left DLPFC using a 70 mm figure-of-8 coil powered by two Magstim 200 stimulators joined with BiStim module. During stimulation the TMS coil was placed over the F5 electrode oriented towards the AF3 electrode. The TMS intensity was determined as the intensity producing a reliable 1 mV electromyogram (EMG) readout at the right abductor pollicis brevis (APB) muscle when stimulation was directed at the motor hot spot.

### TMS-EEG pre-processing

The continuous EEG recordings were segmented into 2 s epochs (1000 ms pre to 1000 ms post TMS). The segments were corrected by the −1000 ms to −100 ms baseline. The segments were trimmed to remove the TMS artifact around −5 ms to 10 ms. Extremely noisy channels (channels removed Pre: 1.96 [0.8] vs Post: 2.2 [1.2]; t-test *p* = 0.256) and epochs (epochs removed Pre: 3.96 [4.3] vs Post: 2.56 [2.6]; t-test *p* = 0.08) were removed through visual inspection. TMS associated decay was removed with the aid of independent component analysis (ICA). The data was filtered with 1–80 Hz bandpass and 58–61 Hz notch. ICA decomposition was computed over the filtered data for a second time to remove other muscle and ocular related artifacts (ICA components removed Pre: 6.2 [1.9] vs Post: 5.57 [2.26]; t-test *p* = 0.11). Finally, Removed electrodes were interpolated, and the data was average referenced. TMS evoked potential (TEP) over the channels domain is shown in Supplemental Figure S1 (−50 ms to 100 ms time window to allow assessment of the activation timing relevant for the current analysis) and Figure S2 (–50 ms to 350 ms time window to allow assessment of the TEP standard, highly replicable waveform).

### Source Localization Procedure

TEP source analysis was executed, cortical, and subcortical (hippocampal) regions were segmented over an ICBM152 generic brain with 15,000 voxels as previously published^47–50^ using the Brainstorm MATLAB toolbox^51^. The EEG cap used in the experiment, Neuroscan 64-channel Quick-Cap, was co-registered to the ICBM152 head model. The geometric head model was computed using the OpenMEEG approach with solution space limited to the cortex and hippocampus^49^. The pre-stimulus period was used to calculate the noise covariance matrix. Finally, the inverse solution was computed based on the sLORETA algorithm^52^ with dipoles constrained to the cortex and hippocampal surface. For each subject, the source localization procedure generated a 15,000 vertices current density map in brain space for each TEP time point.

***Significant current density (SCD) and significant current scatter (SCS)*** was calculated based on equation 1 and 2 (respectively) adapted from methods previously published by Casali and colleagues^37^.

1 $$SCD = SS\left( {x,t} \right) \cdot j\left( {x,t} \right)$$

2 $$SCS = SS\left( {x,t} \right) \cdot d\left( {x - x_{stim}} \right)$$

*SS* (*x,t*) a logical matrix annotating significant sources for each region of interest (i.e., SGC and Hipp) in the brain source space over time. *j* (*x,t*) Instantaneous electrical activity over the head model space. *d* (*x*−*x*_*stim*_) is the distance of every voxel from site of stimulation (i.e., F5). The SS matrix was computed by comparing a permuted distribution of post-stimulus with a permuted distribution of a pre-stimulus per time sample (producing 1000 permutations for each time sample over the epoch distribution, using the Monte-Carlo approach). A current density value for each time sample per voxel was considered significant for *p*-values smaller than 0.05, corrected using false discovery rate (FDR) approach implemented by Matlab^53^.

**Fig. 1:**
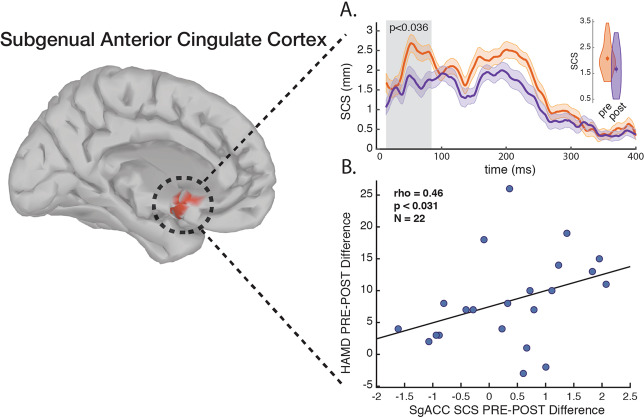
SCS between the DLPFC and the SGC. **a** Pre-MST vs. post-MST trial SCS dynamics, greyed area means are illustrated as inset violin plots. **b** SGC SCS pre-post change correlated with HRSD-24 pre-post change. Time-course shades represent ± SEM.

**Fig. 2:**
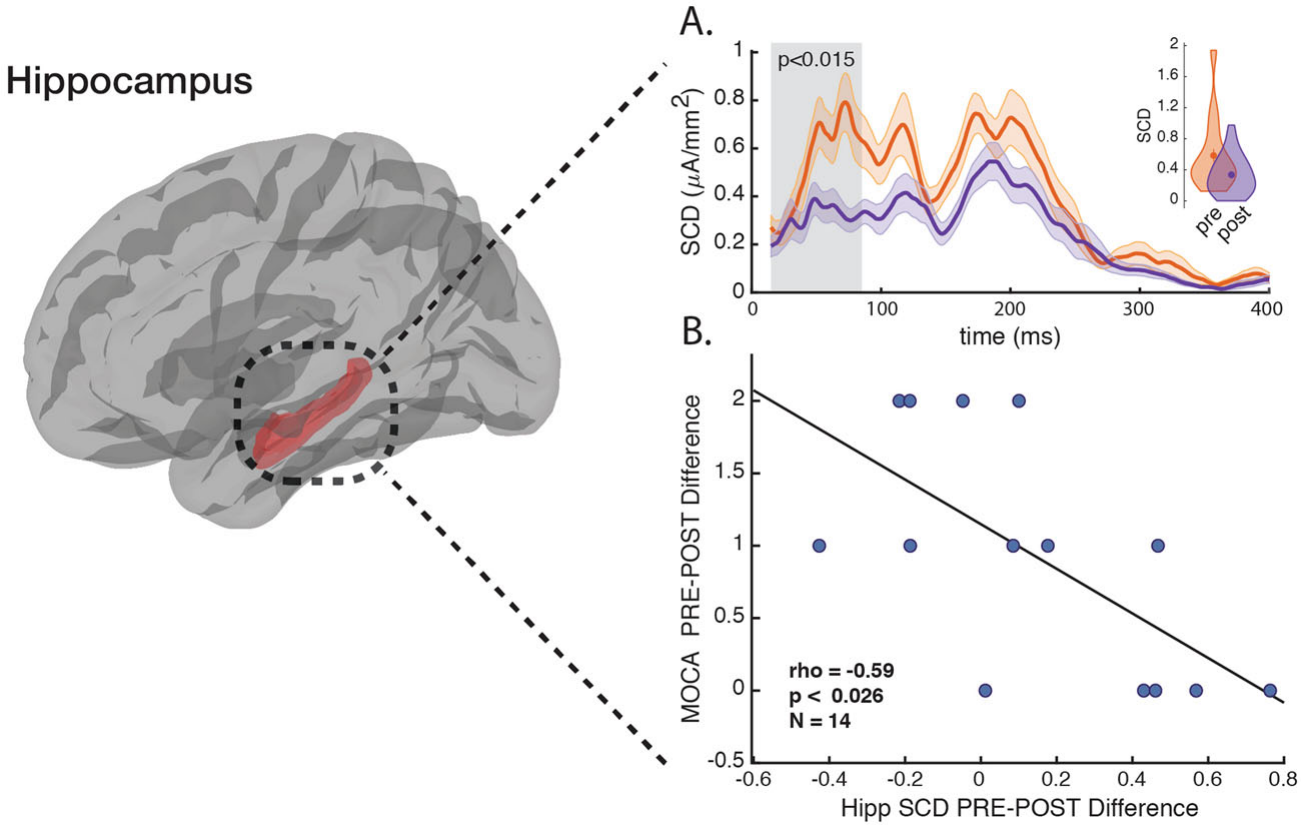
SCD at the Hipp. **a** Pre-MST vs. post-MST trial SCD dynamics, greyed area means are illustrated as inset violin plots. **b** Hipp SCD pre-post change is correlated with MOCA pre-post change. Time-course shades represent ± SEM.

SCD activation and SCS distances were averaged across the TEP significant source activations 15–85 ms after the TMS pulse. This early time window was chosen to avoid controversial TEP timings, which are argued to be artefactual and not immediately caused by the TMS induced effective field itself. The timecourse figures (Figs. 1b and 2b.) show the metric dynamics from 15 ms to 400 ms to illustrate the robustness of the post-MST course effect. The voxel segmentation for the right and left SGC regions as defined by the Destrieux atlas^54^, and for the right and left Hipp regions was segmented using the FreeSurfer atlas^55,56^ with the help of the Brainstorm Matlab toolbox^51^.

### Statistics

To avoid normality assumptions regarding the neurophysiological and clinical scores, pre-post comparisons were made by utilizing the Wilcoxon rank sum test. For the same reasons Spearman correlations were computed to associate the neurophysiological means and the clinical scores per participant (delta of baseline and post-trial values). For some participants clinical values (HRSD-24 and MOCA) were missing at the post trial phase. Extreme outliers (median absolute deviation > 3) were removed from the data. All statistical analyses were made using Matlab version r2017b. All reported values represent mean [±standard deviation].

## Results

Neurophysiology for 31 TRD patients was compared at baseline and following the MST trial in terms of SCS (TMS induced effective connectivity) between the DLPFC and the SGC (Fig. 1), and SCD (TMS induced activation) at the Hipp (Fig. 2).

The SCS between the DLPFC and the SGC is significantly reduced following the MST trial (Pre: 2.0773 [0.5852] mm vs Post: 1.6677 [0.7529] mm; z = 2.097; *p* < 0.036) (Fig. 1b.). The HRSD-24 pre-post difference was correlated significantly with the pre-post difference of the SCS (ρ = 0.46; *P* < 0.031) (Fig. 1c.).

The SCD at the Hipp is significantly reduced following the MST trial (Pre: 0.5837 [0.4601] µA/mm^2^ vs Post: 0.3359 [0.2486] µA/mm^2^; z = 2.436; *p* < 0.015) (Fig. 2b.). The MOCA pre-post difference was correlated significantly with the pre-post difference of the SCD (ρ = −0.59; *P* < 0.026) (Fig. 2c.). However, when looking at MOCA scores for participants included in this study, pre-trial scores compared with post-trial scores did not show significant difference (Pre: 27.57 [2.65] vs Post: 26.64 [2.76]; z = 0.9778; *p* > 0.32).

## Discussion

Using TMS-EEG derived SCS metric we found that the SGC-DLPFC effective connectivity in TRD patients was reduced following MST. This SGC-DLPFC effective connectivity change was associated with HRSD-24 reduction. Using TMS-EEG derived SCD, we also found that the activation at the Hipp was attenuated with MST treatment and that this attenuation of Hipp activation post-trial was correlated with MOCA score reduction.

The SGC-DLPFC relationship has been repeatedly implicated in the pathophysiology and treatment of MDD. This relationship has been reported in different treatment modalities including trials of pharmacotherapy^15^ and neuromodulatory treatments^8,12,13^. The connectivity reduction of the SGC-DLPFC and it’s association with MDD symptom change has been reported across several different methodologies including positron emission tomography (PET)^57–59^ fMRI^8,12,60–62^ and TMS-EEG^13^. Here we utilized a TMS-EEG source localization approach that seem to enable a reliable estimation of the DLPFC-SGC effective connectivity and hippocampal activation changes, following a course of MST. To derive effective connectivity between DLPFC and SGC, we used the TMS-induced metric known as current scattering (SCS). To derive Hipp source activation, we used the TMS-induced metric known as significant source density (SCD).

Our current findings are consistent with previous literature in demonstrating that SGC-DLPFC effective connectivity is attenuated after a course of MST in patients with TRD (Fig. 1a.). This reduction in effective connectivity is correlated with MDD symptom improvement (Fig. 1b.). Additionally, the current connectivity results affirms our previous findings showing DLPFC-SGC effective connectivity attenuation after an rTMS trial for TRD’s^13^.

The activation of the Hipp during ECT is hypothesized to be associated with the treatment related cognitive side effects^30,35,36^. MST treatments appear to have only minimal if any cognitive side effects^34^. The lack of cognitive side effects after MST is believed to be related to the focality of the induced effective field during stimulation^63^. While ECT currents are mainly shunted through the scalp and dispersed throughout cortical and subcortical regions, the MST magnetic pulses pass through the peripheral tissue mostly unimpeded, and induce a relatively focal effective electrical field under the coil^35,36,64,65^. It is hypothesized that wide-spread brain activation during ECT might affect hippocampal structures, which in turn impacts memory function; while MST has a more confined current spread and limited hippocampal activation, thus sparing cognitive functionality^30,35,36^. The complete MST trial cohort and the neurophysiological subset in this study did not show a significant reduction in cognitive function. However, some activation of the Hipp may occur during MST, perhaps only through specific transsynaptic pathways, while ECT might activate the Hipp more broadly though transsynaptic and ephaptic mechanisms^36^. Indeed, we show post-MST Hipp activation changed significantly (Fig. 2a.). Moreover, this change of Hipp activation as measured by the SCD metric was correlated with MOCA score change post trial (Fig. 2b.). This finding supports Hipp association with risk for cognitive side effects after TRD seizure inducing therapy. To further validate this finding, the same TMS-EEG experimental approach should be applied after an ECT trial to determine whether Hipp activation change is consistent with the degree of cognitive side effects.

This study has some limitations. (1) this study concentrate on TRD patients pre and post MST clinical trial. Recruiting healthy control group for evaluating SGC and Hipp neurophysiology was not part of the clinical trial design. However in previous neurophysiological assessment of rTMS trial, we demonstrated how the neuromodulatory treatment normalized the DLPFC-SGC hyper-connectivity to levels similar to healthy controls^13^. (2) The neurophysiological measures and clinical scores may have been influenced by concomitant pharmacotherapies. However, the correlation between the SGC and Hipp neurophysiological changes and changes in clinical scores increases our confidence in the present findings. (3) Targeting the DLPFC with the aid of magnetic resonance imaging (MRI) might have been preferable technique. However, setting-up MRI scans requires time, and delaying the MST treatment for severely depressed patients might prove as a safety issue, due to suicide risk. additionally, it was shown that the F5 electrode is an adequate proxy for DLPFC stimulation^66^. (4) Regarding the source estimations of the Hipp as a subcortical brain structure, although a high-density EEG system is preferable when trying to achieve millimeter scale precision of subcortical activity^67,68^, in this study we explored an a-priori pre-determined, large region of interest that is known to be one of the major drivers of slow-oscillated, high amplitude signal, as registered by EEG^67,69^. The anatomically gross estimation we apply here was performed only pre and post-treatment, and this differential is less sensitive to the above-mentioned methodological nuances. Additionally, the SCD and SCS computations are more robust when dealing with spurious activations and probably more reliable in detecting brain signal. Indeed, the association of SGC significant source metric with depression scores shown here, is replicating a similar finding we previously published for an rTMS study. We strongly believe that the association we found between Hipp activation and cognitive score is reliable. however, we are aware that this finding needs to be replicated in future trials of seizure-inducing technologies for TRD treatment.

## Conclusion

In this study we found two distinct, TRD related neuronal markers: (1) the SGC-DLPFC connectivity marker, which correlated with MDD clinical scores and has been extensively replicated in the literature, under different treatment modalities and by utilizing several experimental settings. (2) The association of the Hipp activation with cognitive adverse effect post convulsive treatment is repeatedly hypothesized in the literature. However, this study provides for the first time, direct evidence for this hypothesis. These markers are a first step in being able to assess two dissociable measures for treatment efficacy and side-effects after an MST trial, and possibly after seizure-inducing treatment application in clinical settings.

## Supplementary information

Supplemental material

Supplemental Figure 1

Supplemental Figure 2

## References

[1] Lepine JP, Briley M (2011). The increasing burden of depression. Neuropsychiatr. Treat..

[2] Patten SB (2009). Canadian network for mood and anxiety treatments (CANMAT) clinical guidelines for the management of major depressive disorder in adults. I. Classification, burden and principles of management. J. Affect Disord..

[3] Rush AJ (2006). Acute and longer-term outcomes in depressed outpatients requiring one or several treatment steps: a STAR*D Report. Am. J. Psychiatry.

[4] Lisanby SH (2007). Electroconvulsive therapy for depression. N. Engl. J. Med..

[5] Drevets WC, Bogers W, Raichle ME (2002). Functional anatomical correlates of antidepressant drug treatment assessed using PET measures of regional glucose metabolism. Eur. Neuropsychopharmacol..

[6] Drevets WC (1997). Subgenual prefrontal cortex abnormalities in mood disorders. Nature.

[7] Mayberg HS (1999). Reciprocal limbic-cortical function and negative mood: converging PET findings in depression and normal sadness. Am. J. Psychiatry.

[8] Fox MD, Buckner RL, White MP, Greicius MD, Pascual-Leone A (2012). Efficacy of TMS targets for depression is related to intrinsic functional connectivity with the subgenual cingulate. Biol. Psychiatry.

[9] Drysdale A. T. et al. Resting-state connectivity biomarkers define neurophysiological subtypes of depression. *Nat Med* 2016; **advance online publication**. 10.1038/nm.4246. 10.1038/nm.4246 PMC5624035 27918562

[10] Weigand A (2018). Prospective validation that subgenual connectivity predicts antidepressant efficacy of transcranial magnetic stimulation sites. Biol. Psychiatry.

[11] McMullen DP (2018). Where to target? The precision medicine approach to brain stimulation. Biol. Psychiatry.

[12] Cash R. F. H. et al. Subgenual functional connectivity predicts antidepressant treatment response to transcranial magnetic stimulation: independent validation and evaluation of personalization. *Biol. Psychiatry*. 10.1016/j.biopsych.2018.12.002 (2019). 10.1016/j.biopsych.2018.12.002 30670304

[13] Hadas I (2019). Association of repetitive transcranial magnetic stimulation treatment with subgenual cingulate hyperactivity in patients with major depressive disorder: a secondary analysis of a randomized clinical trial. JAMA Netw. Open.

[14] Tripp A, Kota RS, Lewis DA, Sibille E (2011). Reduced somatostatin in subgenual anterior cingulate cortex in major depression. Neurobiol. Dis..

[15] Mayberg HS (2000). Regional metabolic effects of fluoxetine in major depression: serial changes and relationship to clinical response. Biol. Psychiatry.

[16] Kennedy SH (2001). Changes in regional brain glucose metabolism measured with positron emission tomography after paroxetine treatment of major depression. Am. J. Psychiatry.

[17] Kennedy SH (2007). Differences in brain glucose metabolism between responders to CBT and venlafaxine in a 16-week randomized controlled trial. Am. J. Psychiatry.

[18] Keedwell P (2009). Neural markers of symptomatic improvement during antidepressant therapy in severe depression: subgenual cingulate and visual cortical responses to sad, but not happy, facial stimuli are correlated with changes in symptom score. J. Psychopharmacol..

[19] Mottaghy FM (2002). Correlation of cerebral blood flow and treatment effects of repetitive transcranial magnetic stimulation in depressed patients. Psychiatry Res Neuroimaging.

[20] Liston C (2014). Default mode network mechanisms of transcranial magnetic stimulation in depression. Biol. Psychiatry.

[21] Nobler MS (2001). Decreased regional brain metabolism after ECT. Am. J. Psychiatry.

[22] Mayberg HS (2005). Deep brain stimulation for treatment-resistant depression. Neuron.

[23] Argyelan M (2016). Subgenual cingulate cortical activity predicts the efficacy of electroconvulsive therapy. Transl. Psychiatry.

[24] Leaver AM (2016). Modulation of intrinsic brain activity by electroconvulsive therapy in major depression. Biol. Psychiatry Cogn. Neurosci. Neuroimaging.

[25] Lyden H (2014). Electroconvulsive therapy mediates neuroplasticity of white matter microstructure in major depression. Transl. Psychiatry.

[26] Redlich R (2016). Prediction of individual response to electroconvulsive therapy via machine learning on structural magnetic resonance imaging data. JAMA Psychiatry.

[27] Sackeim, H. A. Memory and ECT From Polarization to reconciliation. J. ECT 16, 87 (2000). 10.1097/00124509-200006000-00001 10868319

[28] Moscrip TD, Terrace HS, Sackeim HA, Lisanby SH (2004). A primate model of anterograde and retrograde amnesia produced by convulsive treatment. J. ECT.

[29] Aoki Y (2016). The experience of electroconvulsive therapy and its impact on associated stigma: a meta-analysis. Int J. Soc. Psychiatry.

[30] Kallioniemi E, McClintock SM, Deng Z-D, Husain MM, Lisanby SH (2019). Magnetic seizure therapy: towards personalized seizure therapy for major depression. Pers. Med Psychiatry.

[31] Schlaepfer TE, Lisanby HS, Fisch H-U, Sackeim HA (2002). Magnetic seizure induction for the treatment of major depression. Eur. Psychiatry.

[32] Lisanby SH, Luber B, Schlaepfer TE, Sackeim HA (2003). Safety and feasibility of magnetic seizure therapy (MST) in major depression: randomized within-subject comparison with electroconvulsive therapy. Neuropsychopharmacology.

[33] Fitzgerald PB (2018). A pilot study of the comparative efficacy of 100 Hz magnetic seizure therapy and electroconvulsive therapy in persistent depression. Depress Anxiety.

[34] Daskalakis Z. J. et al. Magnetic seizure therapy (MST) for major depressive disorder. *Neuropsychopharmacology* 2019; 1–7. 10.1038/s41386-019-0515-4 PMC6901571 31486777

[35] Lisanby SH (2002). Update on magnetic seizure therapy: a novel form of convulsive therapy. J. ECT.

[36] Lisanby S. H. et al. Chapter 9 Neurophysiological characterization of magnetic seizure therapy (MST) in non-human primates. In: Paulus W. et al. (eds). *Supplements to Clinical Neurophysiology*. Elsevier, 2003, pp 81–99. 10.1016/s1567-424x(09)70212-0 14677385

[37] Casali A. G., Casarotto S., Rosanova M., Mariotti M., Massimini M. General indices to characterize the electrical response of the cerebral cortex to TMS. *NeuroImage* 2010; **49**: 1459–1468. 10.1016/j.neuroimage.2009.09.026 19770048

[38] Biabani M., Fornito A., Mutanen T. P., Morrow J. & Rogasch N. C. Characterizing and minimizing the contribution of sensory inputs to TMS-evoked potentials. *Brain Stimulat*. 10.1016/j.brs.2019.07.009 (2019). 10.1016/j.brs.2019.07.009 31377097

[39] Conde V (2019). The non-transcranial TMS-evoked potential is an inherent source of ambiguity in TMS-EEG studies. NeuroImage.

[40] Freedberg M, Reeves JA, Hussain SJ, Zaghloul KA, Wassermann EM (2020). Identifying site- and stimulation-specific TMS-evoked EEG potentials using a quantitative cosine similarity metric. PLoS ONE.

[41] Massimini M (2005). Breakdown of cortical effective connectivity during sleep. Science.

[42] Casali A. G. et al. A theoretically based index of consciousness independent of sensory processing and behavior. *Sci Transl Med* 2013; **5**: 198ra105-198ra105. 10.1126/scitranslmed.3006294 23946194

[43] Ferrarelli F (2010). Breakdown in cortical effective connectivity during midazolam-induced loss of consciousness. Proc. Natl Acad. Sci. USA.

[44] Ferrarelli F, Riedner BA, Peterson MJ, Tononi G (2015). Altered prefrontal activity and connectivity predict different cognitive deficits in schizophrenia: prefrontal deficits in schizophrenia patients. Hum. Brain Mapp..

[45] Casarotto S (2011). Transcranial magnetic stimulation-evoked EEG/cortical potentials in physiological and pathological aging. NeuroReport.

[46] Johnson JS, Kundu B, Casali AG, Postle BR (2012). Task-dependent changes in cortical excitability and effective connectivity: a combined TMS-EEG study. J. Neurophysiol..

[47] Attal Y. et al. Modeling and detecting deep brain activity with MEG EEG. in *2007 29**th Annual International Conference of the IEEE Engineering in Medicine and Biology Society*. 2007, pp 4937–4940. 10.1109/IEMBS.2007.4353448 18003114

[48] Attal Y, Schwartz D (2013). Assessment of subcortical source localization using deep brain activity imaging model with minimum norm operators: a MEG study. PLOS ONE.

[49] Chupin M (2007). Anatomically constrained region deformation for the automated segmentation of the hippocampus and the amygdala: Method and validation on controls and patients with Alzheimer’s disease. NeuroImage.

[50] Dumas T (2013). MEG evidence for dynamic amygdala modulations by gaze and facial emotions. PLoS ONE.

[51] Tadel F., Baillet S., Mosher J. C., Pantazis D. & Leahy R. M. Brainstorm: a user-friendly application for MEG/EEG analysis. *Comput Intell Neurosci*. 10.1155/2011/879716 (2011). 10.1155/2011/879716 PMC3090754 21584256

[52] Pascual-Marqui RD (2002). Standardized low-resolution brain electromagnetic tomography (sLORETA): technical details. Methods Find. Exp. Clin. Pharm..

[53] Benjamini Y, Hochberg Y (1995). Controlling the false discovery rate: a practical and powerful approach to multiple testing. J. R. Stat. Soc. Ser. B Methodol..

[54] Destrieux C, Fischl B, Dale A, Halgren E (2010). Automatic parcellation of human cortical gyri and sulci using standard anatomical nomenclature. NeuroImage.

[55] Fischl B (2004). Sequence-independent segmentation of magnetic resonance images. NeuroImage.

[56] Fischl B (2002). Whole brain segmentation: automated labeling of neuroanatomical structures in the human brain. Neuron.

[57] Teneback CC (1999). Changes in prefrontal cortex and paralimbic activity in depression following two weeks of daily left prefrontal TMS. J. Neuropsychiatry Clin. Neurosci..

[58] Nahas Z (2001). Brain effects of TMS delivered over prefrontal cortex in depressed adults. J. Neuropsychiatry Clin. Neurosci..

[59] Kito S, Fujita K, Koga Y (2008). Regional cerebral blood flow changes after low-frequency transcranial magnetic stimulation of the right dorsolateral prefrontal cortex in treatment-resistant depression. Neuropsychobiology.

[60] Zhou Y (2010). Increased neural resources recruitment in the intrinsic organization in major depression. J. Affect Disord..

[61] Downar J (2014). Anhedonia and reward-circuit connectivity distinguish nonresponders from responders to dorsomedial prefrontal repetitive transcranial magnetic stimulation in major depression. Biol. Psychiatry.

[62] Salomons TV (2014). Resting-state cortico-thalamic-striatal connectivity predicts response to dorsomedial prefrontal rTMS in major depressive disorder. Neuropsychopharmacology.

[63] McClintock SM, Tirmizi O, Chansard M, Husain MM (2011). A systematic review of the neurocognitive effects of magnetic seizure therapy. Int Rev. Psychiatry.

[64] Deng Z.-D., Lisanby S. H. & Peterchev A. V. Effect of anatomical variability on neural stimulation strength and focality in electroconvulsive therapy (ECT) and magnetic seizure therapy (MST). in *2009 Annual International Conference of the IEEE Engineering in Medicine and Biology Society*. 2009, pp 682–688. 10.1109/IEMBS.2009.5334091 19964484

[65] Deng Z-D, Lisanby SH, Peterchev AV (2011). Electric field strength and focality in electroconvulsive therapy and magnetic seizure therapy: a finite element simulation study. J. Neural Eng..

[66] Rusjan PM (2010). Optimal transcranial magnetic stimulation coil placement for targeting the dorsolateral prefrontal cortex using novel magnetic resonance image-guided neuronavigation. Hum. Brain Mapp..

[67] Seeber M (2019). Subcortical electrophysiological activity is detectable with high-density EEG source imaging. Nat. Commun..

[68] Song J (2015). EEG source localization: sensor density and head surface coverage. J. Neurosci. Methods.

[69] Buzsáki G (2002). Theta oscillations in the hippocampus. Neuron.

