# Supplementary material for: Subgenual cingulate connectivity and hippocampal activation are related to MST therapeutic and adverse effects
Source: Transl Psychiatry. 2020 Nov 10;10:392. doi: 10.1038/s41398-020-01042-7 (PMC7655940; doi:10.1038/s41398-020-01042-7)

**List of supporting material:**

1. Figure S1: TMS evoked potential (TEP) over the channels domain at -50 ms to 100 ms time window.
2. Figure S2: TMS evoked potential (TEP) over the channels domain at -50 ms to 350 ms time window.

**Supporting material:**

**Figure S1.** TMS evoked potential (TEP) over the channels domain at -50 ms to 100 ms time window.


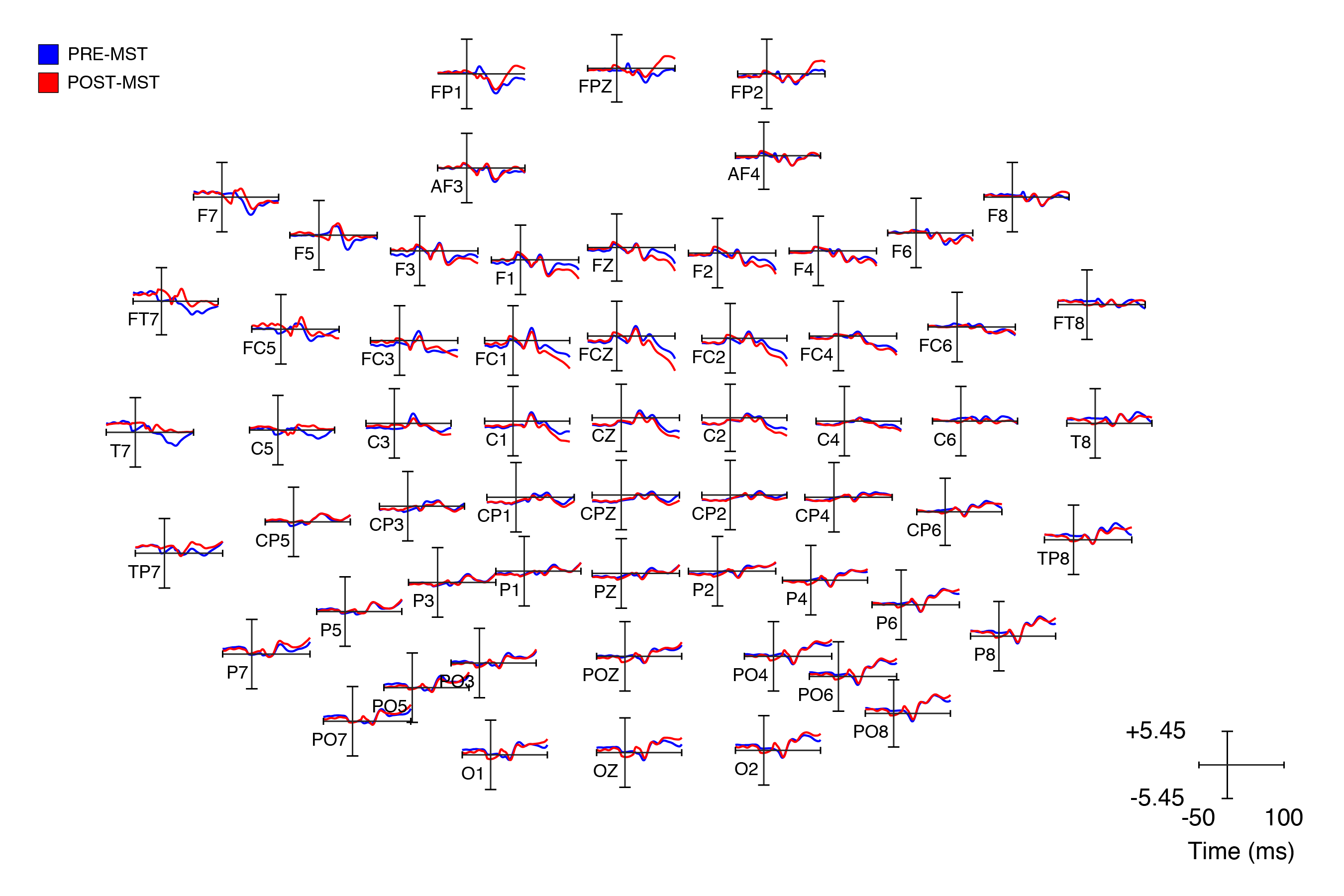


**Figure S2.** TMS evoked potential (TEP) over the channels domain at -50 ms to 350 ms time window.


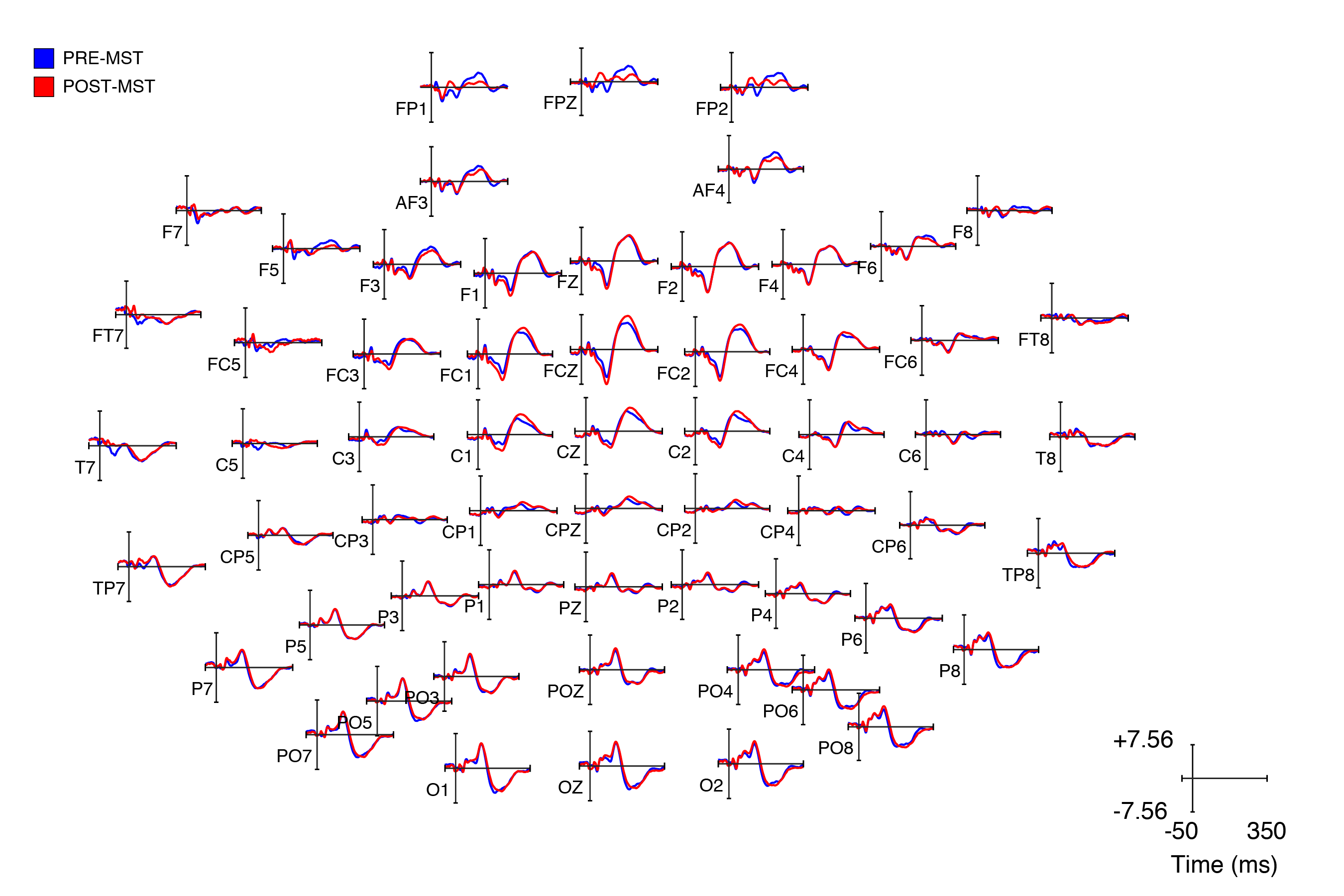

Supplement: Supplementary file 1 — Supplemental material [file 41398_2020_1042_MOESM1_ESM.docx]
