# Supplementary figures and images for: Subgenual cingulate connectivity and hippocampal activation are related to MST therapeutic and adverse effects
Source: Transl Psychiatry. 2020 Nov 10;10:392. doi: 10.1038/s41398-020-01042-7 (PMC7655940; doi:10.1038/s41398-020-01042-7)

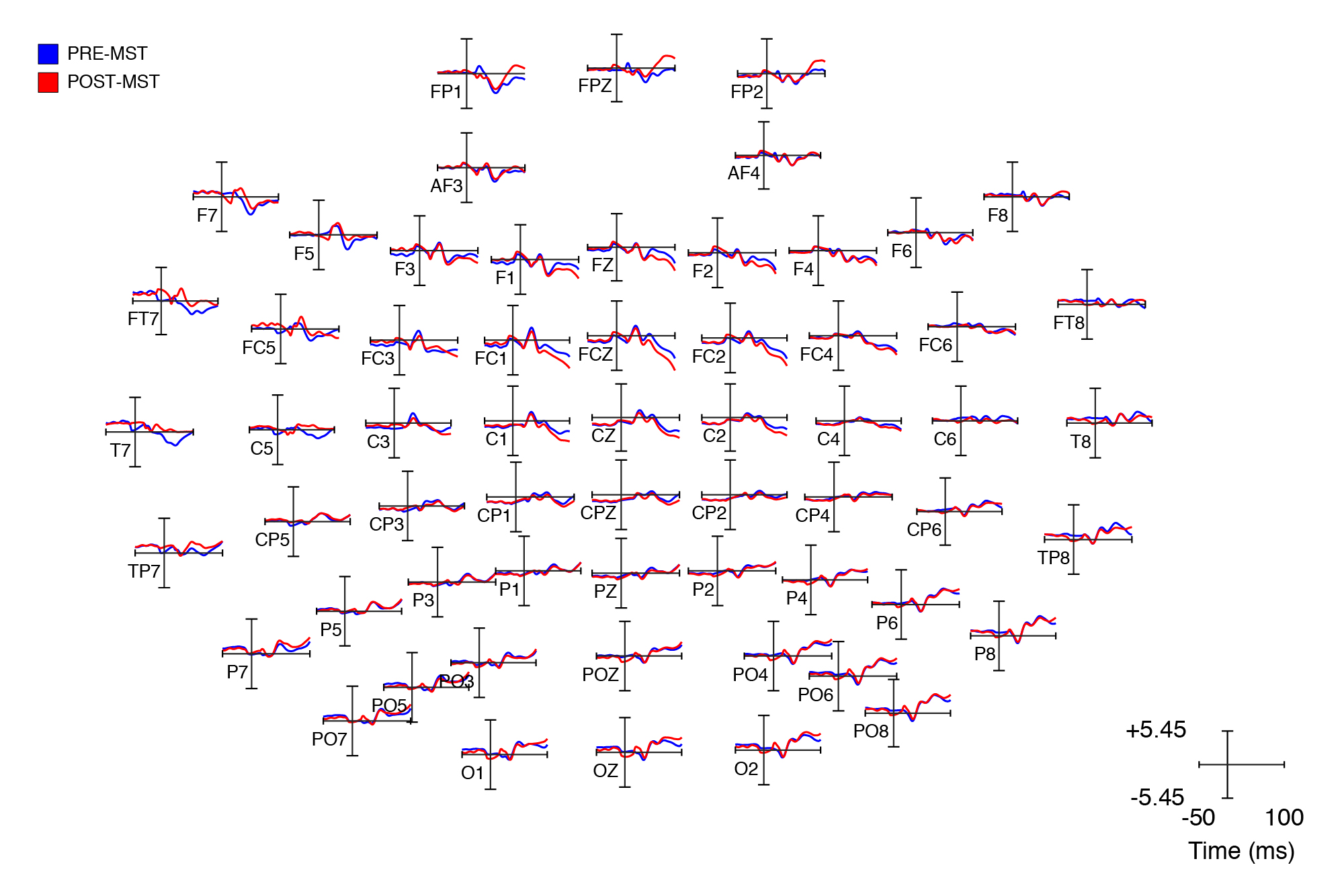

Supplement: Supplementary file 2 — Supplemental Figure 1 [file 41398_2020_1042_MOESM2_ESM.jpg]

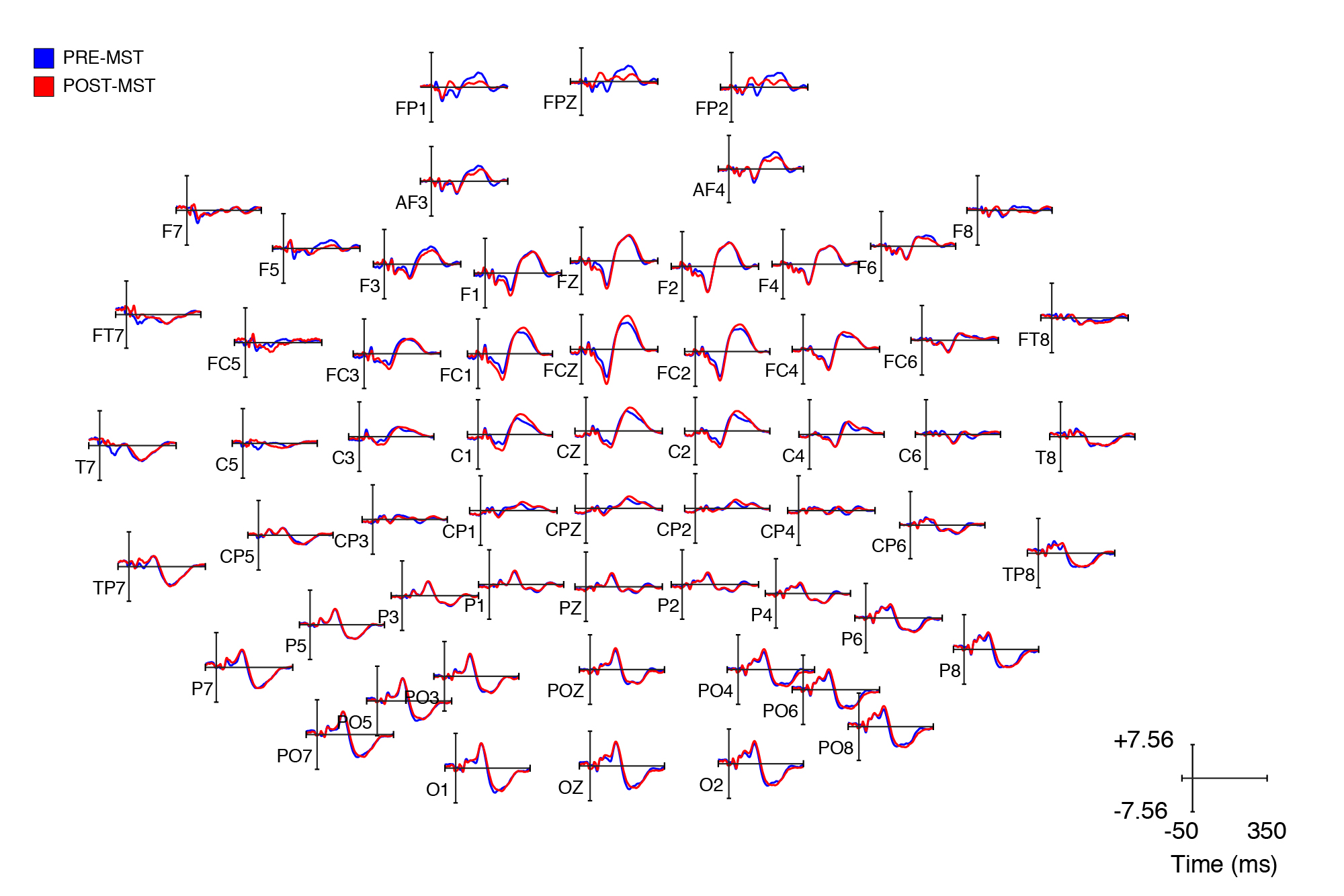

Supplement: Supplementary file 3 — Supplemental Figure 2 [file 41398_2020_1042_MOESM3_ESM.jpg]
